# Probable Autoimmune Depression in a Patient With Multiple Sclerosis and Antineuronal Antibodies

**DOI:** 10.3389/fpsyt.2020.00745

**Published:** 2020-08-13

**Authors:** Dominique Endres, Sebastian Rauer, Nils Venhoff, Patrick Süß, Rick Dersch, Kimon Runge, Bernd L. Fiebich, Kathrin Nickel, Miriam Matysik, Simon Maier, Katharina Domschke, Karl Egger, Harald Prüss, Ludger Tebartz van Elst

**Affiliations:** ^1^Section for Experimental Neuropsychiatry, Department of Psychiatry and Psychotherapy, Medical Center-University of Freiburg, Faculty of Medicine, University of Freiburg, Freiburg, Germany; ^2^Department of Psychiatry and Psychotherapy, Medical Center-University of Freiburg, Faculty of Medicine, University of Freiburg, Freiburg, Germany; ^3^Department of Neurology, Medical Center-University of Freiburg, Faculty of Medicine, University of Freiburg, Freiburg, Germany; ^4^Department of Rheumatology and Clinical Immunology, Medical Center-University of Freiburg, Faculty of Medicine, University of Freiburg, Freiburg, Germany; ^5^Department of Molecular Neurology, University Hospital Erlangen, Erlangen, Germany; ^6^Center for Basics in Neuromodulation, Faculty of Medicine, University of Freiburg, Freiburg, Germany; ^7^Department of Neuroradiology, Medical Center-University of Freiburg, Faculty of Medicine, University of Freiburg, Freiburg, Germany; ^8^Department of Neurology and Experimental Neurology, Charité – Universitätsmedizin Berlin, Berlin, Germany; ^9^German Center for Neurodegenerative Diseases (DZNE), Berlin, Germany

**Keywords:** depression, multiple sclerosis, connective tissue disease, autoimmune encephalitis, autoantibody

## Abstract

**Background:**

In a subgroup of patients with mood disorders, clear-cut organic disorders are responsible for depressive symptoms (e.g., autoimmune diseases such as multiple sclerosis or systemic lupus erythematosus). In these cases, an organic affective disorder can be diagnosed.

**Case Presentation:**

The authors present the case of a 59-year-old male patient who developed a severe depressive episode over approximately 6 months and was, therefore, admitted to the hospital. In retrospect, he reported that, at age 39, he suffered from self-limiting sensory disturbances and muscle weakness in both legs. The current magnetic resonance imaging of his brain showed several conspicuous FLAIR-hyperintense supratentorial white matter lesions compatible with chronic inflammatory brain disease. Imaging of the spinal axis revealed no clear spinal lesions. Cerebrospinal fluid (CSF) analyses showed CSF-specific oligoclonal bands. Therefore, multiple sclerosis was diagnosed. Further CSF analyses, using tissue-based assays with indirect immunofluorescence on unfixed murine brain tissue, revealed a (peri-)nuclear signal and a strong neuritic signal of many neurons, especially on granule cells in the cerebellum, hippocampus, and olfactory bulb, as well as in the corpus callosum. Additionally, antinuclear antibody (ANA) titers of 1:12,800 and a lymphopenia were detected in blood tests. Further system clarification showed no suspicion of rheumatic or oncological disease. Anti-inflammatory treatment led to rapid and sustained improvement.

**Conclusion:**

The present patient suffered from a probable “autoimmune depression” in the context of newly diagnosed multiple sclerosis with typical MRI and CSF pathologies, alongside mild concomitant latent systemic autoimmune process (with high-titer ANAs and lymphopenia) and unknown antineuronal antibodies. The case report illustrates that a depressive syndrome suggestive of primary idiopathic depressive disorder may be associated with an autoimmune brain involvement. The detection of such organic affective disorders is of high clinical relevance for affected patients, as it enables alternative and more causal treatment approaches.

## Background

Mood disorders are one of the most common mental illnesses, and they are the most limiting factors regarding quality of life. In a small subgroup of patients with depressive episodes, organic disorders are responsible for depressive symptoms (e.g., multiple sclerosis, systemic lupus erythematosus, stroke, and hypothyroidism). In these cases, an organic affective disorder can be diagnosed (1). In particular, a number of autoimmune diseases with central nervous system (CNS) involvement can lead to affective symptoms (2–4). Depressive symptoms occur in about 50% of patients with multiple sclerosis (5). However, they can also occur in the context of different connective tissue diseases, especially in systematic lupus erythematosus (SLE) (6, 7). Predominant brain involvement of SLE refers to neuropsychiatric SLE (8, 9). However, affective symptoms can also occur in the context of Hashimoto encephalopathy (4, 10) or other autoimmune encephalitides, such as anti-NMDA receptor encephalitis (11). Most of these autoimmune syndromes are associated with neuropsychiatric symptoms (e.g., focal neurological deficits in multiple sclerosis or epileptic seizures in autoimmune encephalitis) or other organ involvement (e.g., joint involvement in SLE) (2, 4, 12). The extent to which isolated depressive syndromes are caused by clear autoimmune pathophysiology is still largely unknown. Various blood tests, including the measurement of antineuronal autoantibodies, electroencephalography (EEG), magnetic resonance imaging (MRI), [^18^F]-fluorodeoxyglucose positron emission tomography (FDG-PET), and cerebrospinal fluid (CSF) diagnostics may contribute to the detection of an autoimmune disorder of the CNS (13). The rationale of this article is to present a patient with probable “autoimmune depression”.

## Case Presentation

Here, the authors present the case of a 59-year-old male patient who, over approximately 6 months, developed a severe depressive episode with depressed mood, loss of interest, reduced energy, reduced concentration and attention, pessimistic views of the future, disturbed sleep, and distressing inner restlessness. The psychopharmacological treatment with sertraline, trimipramine, trazodone, and cognitive behavioral therapy did not lead to an improvement, which is why the patient was admitted to our psychiatric day-care hospital. Focal neurological symptoms or other general medical symptoms or signs (e.g., skin changes) were not present. The patient had already experienced one mild depressive episode when he was 55 years old. The possibility of multiple sclerosis had already been discussed at the age of 39. At that time, he had suffered from sensory disturbances and muscle weakness of both legs (emphasized on the right side). Already at that time, CSF-specific oligoclonal bands (OCBs) and MRI white matter (WM) lesions had been noticed. However, with clinical symptoms fading away (without treatment) those MRI images had got lost over the years. When the patient was 44, autoimmune hepatopathy had been discussed due to slightly elevated transaminases, evidence of fatty liver in abdominal ultrasound, and elevated antinuclear antibodies (ANAs; titer: 1:3,200; reference, <1:50) without specification for extractable nuclear antigens (ENAs).

### Diagnostic Findings

**Figure 1:**
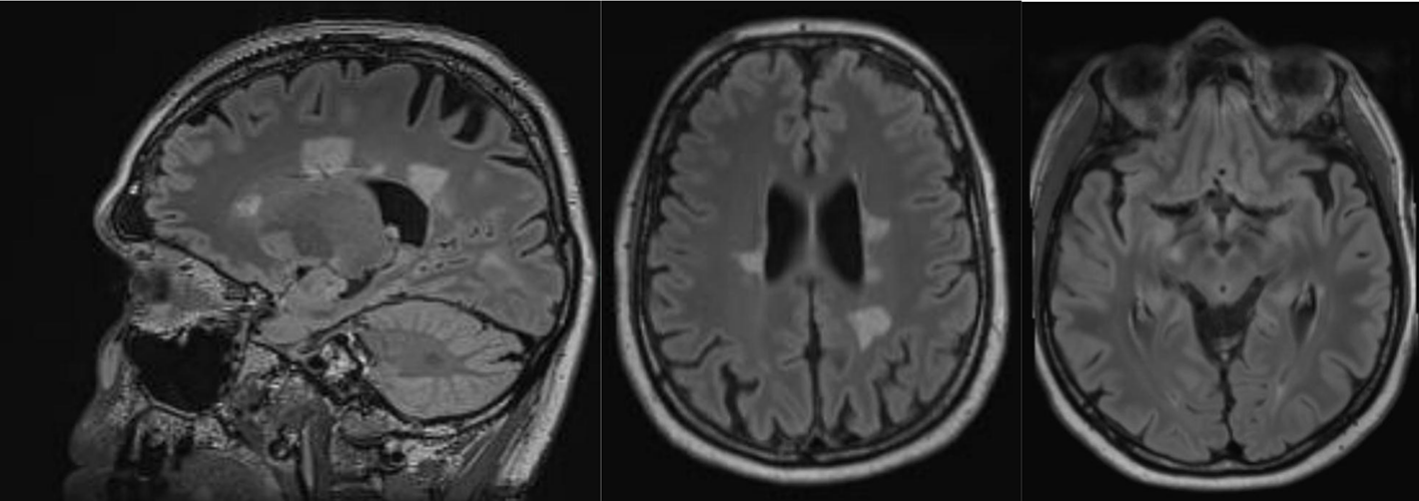
Magnetic resonance imaging showed conspicuous supratentorial white matter lesions without contrast enhancement. This is compatible with chronic inflammatory brain disease.

**Figure 2:**
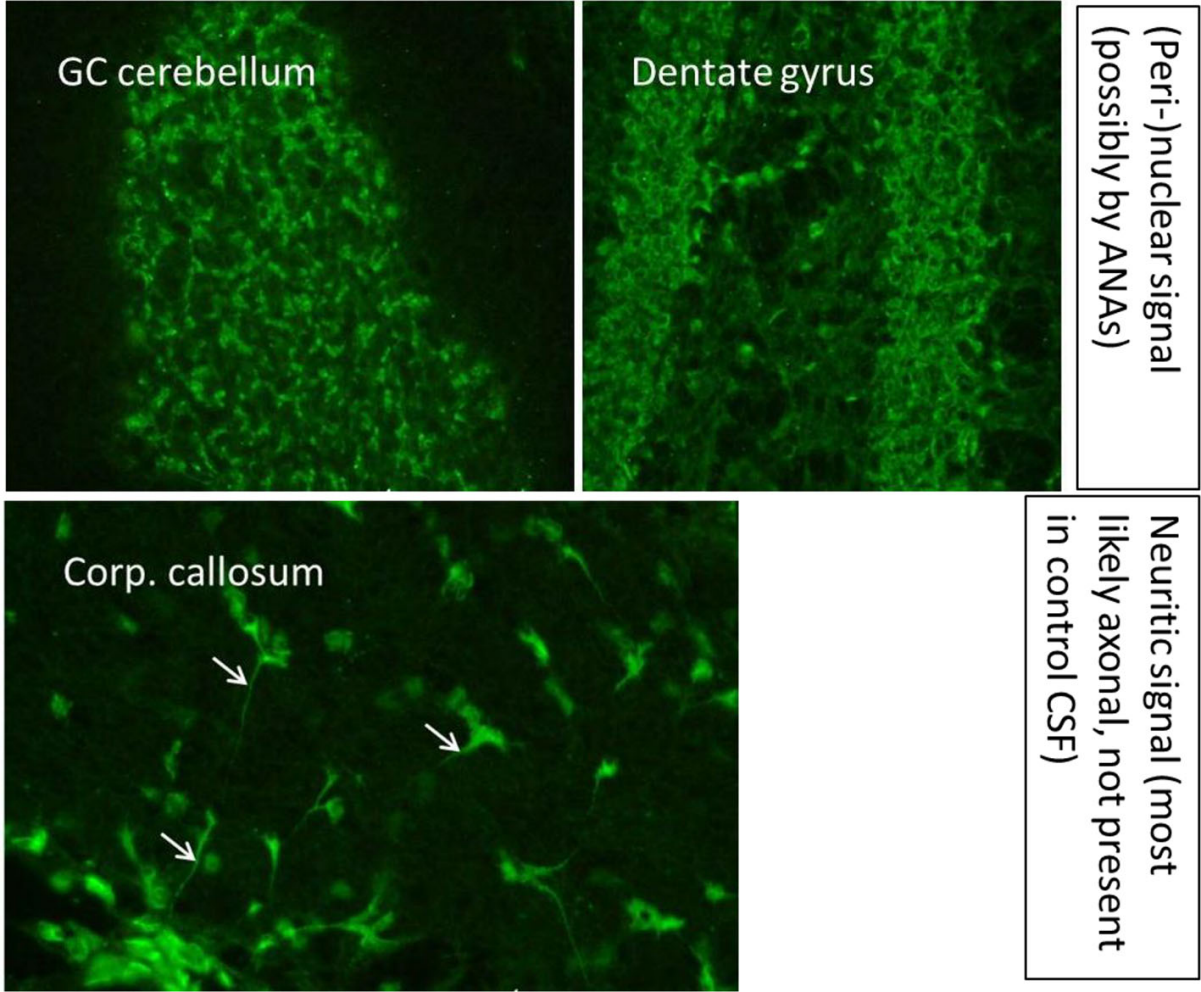
A tissue-based assay using indirect immunofluorescence screening on brain sections of rodents showed binding of especially cerebrospinal fluid antibodies to granule cells in the granule cell layer of the cerebellum and the hippocampus and in the corpus callosum. Methodological aspects of the antibody test are described in Kreye et al. (14) and Edwin Thanarajah et al. (15). ANA, antinuclear antibodies; CSF, cerebrospinal fluid; GC, granule cell; Corp., corpus.

An MRI of the neurocranium showed several conspicuous FLAIR-hyperintense supratentorial WM lesions (among others, ovoid periventricular WM lesions on both sides, in the corpus callosum, and in the right side of the crus cerebri) without contrast enhancement which was assessed to be compatible with chronic inflammatory brain disease (**Figure 1**). Imaging of the spinal axis revealed no clear spinal lesions. Serum ANA titers of 1:12,800 (reference: <1:50) were found using indirect immunofluorescence technique on HEp-2000^®^ cells without specification for ENAs or double-stranded DNA (ds-DNA). The CSF diagnostics revealed CSF-specific OCBs and local immunoglobulin M (IgM) synthesis. The MRZ reaction was negative. A large panel of established antineuronal antibodies against cell surface (NMDA-R, LGI1, CASPR2, AMPA1/2-R, GABA-B-R, and DPPX), and intracellular (Yo, Hu, CV2/CRMP5, Ri, Ma1/2, Tr, Zic4, SOX1, GAD65, and amphiphysin) antigens were negative. The tissue-based assay using CSF with indirect immunofluorescence on unfixed murine brain tissue revealed a (peri-)nuclear signal, likely reflecting the ANAs. However, CSF-testing also displayed an arborizing neuronal signal of many neurons, especially on granule cells in the cerebellum, hippocampus and olfactorius bulb (most likely axonal, not present in control CSF). The neuronal signal was also detectable in the corpus callosum (**Figure 2**). This signal was less detectable in the serum. The EEG and FDG PET of the brain and the whole body showed no relevant abnormalities. The peripheral electrophysiological examinations were essentially normal. Comprehensive blood analyses revealed persistent lymphopenia (minimum 0.59 10^9^/L; reference 1.1–3.2 10^9^/L). The lymphocyte panel showed a deficiency of CD8+ T cells, and the CD4/CD8 quotient was elevated (6.92), however the sarcoidosis markers were unremarkable. Further medical investigations including gastroscopy, coloscopy, ultrasound of the abdomen, and an x-ray of the thorax did not produce any evidence for rheumatic or oncological disease. Neuropsychologically, a clear psychomotor slowing and deficits in verbal memory were observed. All diagnostic findings are summarized in **Table 1**.

**Table 1:** Diagnostic findings performed during the patient’s day clinic stay, 6 months after the onset of symptoms.

**Blood analyses**	•Normal electrolytes, C reactive protein not increased, normal lipase, **slightly increased GPT** (63 U/l; referenc, 10–50 U/L). Blood cellcount showed **lymphopenia** throughout (minimum 0.59 Tsd/µl; reference, 1.1–3.2 10^9^/L). •**Vitamin B12** (168 pg/ml; reference, 197–771 pg/ml), **folid acid** (2.6 ng/ml; reference, 5.6–45.8 ng/ml), and **selenium** (68 μg/l; reference, 75–140 μg/l) were decreased. **Vitamin D** was not optimal (21.9 ng/ml; optimal: >30 ng/ml). •Thyroid-stimulating hormone, triiodothyronine, and thyroxine levels were in normal ranges. Autoantibodies against thyroglobulin, TSH receptor, and thyroid peroxidase were not increased. •Serologies for Lyme borreliosis, syphilis, and HIV were negative. •No antibodies against the intracellular onconeural antigens Yo, Hu, CV2/CRMP5, Ri, Ma1/2, SOX1, or the intracellular synaptic antigens GAD65/amphiphysin were found (using Ravo line assay^®^). •Antibodies against different neuronal cell surface antigens (NMDA-R, AMPA-R, GABA-B-R, DPPX, VGKC-complex [LGI1, Caspr2]) were negative (using Euroimmun biochip-assay^®^). •**Tissue-based assay with indirect immunofluorescence (IF) on unfixed murine brain tissue showed a slight (peri-)nuclear signal, but also a neuronal arborized signal of many neurons, especially granule cells in the cerebellum and hippocampus as well as in the olfactorius bulb (most likely axonal). The neuronal signal was also detectable in the corpus callosum**. •Aquaporin 4 and MOG antibodies were negative (using Euroimmun assay^®^). •**Screening for antinuclear antibodies (ANA) in IIF showed increased titers (1:12,800; reference, <1:50)** without specification for ENAs (*anti-SnRNP/Sm*, *anti-Sm*, *anti-SS-A/Ro*, *anti-Ro-52*, *anti-SS-A/Ro*, *anti-Ro-52*, *anti-SS-B/La*, *anti-Scl-70*, *anti-PM-Scl*, *anti-Jo-1*, *anti-centromere*, *anti-PCNA*, *anti-nucleosome*, *anti-histone*, *anti-ribos. P-protein*, *anti-AMA-M2*, *anti-DFS70*, *anti-Mi-2 alpha/beta*, *anti-Ku*, *anti-PM-Scl100*, *anti-Pm-Scl75*, *anti-Jo-1*, *anti-SRP*, *anti-PL-7/12*, *anti-EJ*, *anti-OJ*, *anti-Ro-52*, *anti-Tif1g*, *anti-MDA5*,*anti-NXP2*, *anti-SAE1*) or ds-DNA. Anti-neutrophil cytoplasmic antibodies, antiphospholipid antibodies were not clearly positive (+) without increased anti-MPO and PR3 antibodies. Rheumatoid factor and anti-mitochondrial antibodies were negative. No changes in the complement system (C3, C4, CH50, C3d) were observed. •IgG levels were normal, **IgA was increased** (4.37 g/L; reference, 0.70–4 g/L) and **IgM was decreased** (0.2 g/L, reference 0.4–2.3 g/L); immunofixation showed no monoclonal antibody production. •**T-cell panel showed a deficiency of CD8+ T cells. The CD4/CD8 quotient was elevated (6.92)**. •“Sarcoidosis parameters” (interleukin-2-receptor, neopterin, and ACE) were not increased.
**Cerebrospinal fluid analyses (CSF)**	•No evidence of a malignant process. •Normal white blood cell count (1/µL; reference, <5/µL). •Normal protein concentration (310 mg/L; reference, <450 mg/L), and normal age-corrected albumin quotient: 3.6; age-dependent reference, <8 × 10^–3^). •**CSF specific oligoclonal bands**; IgG index not increased (0.63; reference, ≤0.7). •**Local IgM synthesis** (no longer detectable in the control examination).•CSF lactate not increased (1.87 mmol/L; reference, 1.7–2.6 mmol/L). •Antibodies against neuronal cell surface antigens (*NMDAR*, *AMPA-R*, *GABA-B-R*, *DPPX*, *VGKC-complex [LGI1*, *Caspr2]*) were negative (Euroimmun Biochip assay^®^). •**“Tissue-based assay with indirect immunofluorescence on unfixed murine brain tissue showed a strong (peri-)nuclear signal, but also a neuritic signal of many neurons, especially granule cells in the cerebellum and hippocampus as well as in the olfactorius bulb (most likely axonal). The neuritic signal was also detectable in the corpus callosum**.
**Cerebral magnetic resonance imaging**	•**Several conspicuous FLAIR-hyperintense supratentorial white matter lesions (among others ovally configured on both sides periventricular as well as in the corpus callosum and in the crus cerebri on the right side) without contrast agent uptake, compatible with chronic inflammatory brain disease**.
**Magnetic resonance imaging of the spinal axis**	•No clear spinal lesions (with partial artifact superimposition of the thoracic spine).
**Electroencephalography**	•Normal alpha rhythm, no epileptic pattern or pathological slowing.
**[^18^F]fluorodeoxyglucose positron emission tomography**	•Unsuspicious brain metabolism. •No metabolic changes or structural lesion suspicious of malignancy on whole-body PET/CT. **Low-grade increase in metabolism axillary and inguinal lymph node left pronounced, most likely unspecific**.
**Peripheral electrophysiological investigations**	•Inconspicuous tibialis and medianus SEPs on both sides with inconspicuous suralis neurography on the right. •In the MEPs no indication of impaired efference to the arms and legs.
**Gastroscopy, coloscopy**	•No evidence of malignancy in the upper gastrointestinal tract, **mild, chronic, inactive antral and corpus gastritis. Coloscopy showed a rectal polyp (tubular adenoma with low grade intraepithelial neoplasia)**.
**Sonography of the abdomen**	•**Steatosis hepatis grade II, uncomplicated liver cyst in segment VI, separated gallbladder**.
**X-ray thorax**	•No indication of fibrotic or granulomatous changes. No tumor suspicious round heart.
**Cardiological examinations**	•Normal electrocardiography and transthoracic echocardiography, especially no indication of right heart strain.

### Developmental, Somatic, and Family Histories

The patient’s history was negative for in-utero/birth complications, febrile convulsions/epileptic seizures, severe infectious diseases, or craniocerebral traumata. There were no indications of developmental disorders. An obsessive-compulsive personality structure was perceivable without ever fulfilling the criteria of a personality disorder. The family history (including his parents, grandparents, and siblings) was clear of any diagnosed psychiatric, neurological, cancer, or autoimmune disorders except for his mother suffering from beginning late-onset dementia.

### Treatment and Outcome

Following diagnostic clarification and in the absence of a response to antidepressants, immunosuppressive treatment with a steroid pulse (5 × 1000 mg methylprednisolone over 5 days) was performed. Directly after steroid pulse treatment, the inner restlessness receded, and the mood improved. Within the next two to three weeks, the depressive syndrome fully remitted. Neuropsychological follow-up testing showed an improvement in working memory and mental flexibility, and the ANA titers decreased to 3200. Maintenance therapy with azathioprine led to pancreatitis, therefore maintenance therapy with methotrexate (15 mg per week) was established. The individual decision for methotrexate treatment was made under the initial assumption of a possible “overlap syndrome” with multiple sclerosis and a systemic autoimmune disease. At the time of publication, the patient was recommended to start a long-term, specific treatment of multiple sclerosis. Vitamin B12/D and folic acid were substituted. After improvement of the depression, the antidepressant treatment was tapered off. Three months later, the MRI was unchanged. Eight months later, one small, newly detectable lesion in the right cerebellar peduncle was observed (in the previous MRI examination, however, the area was artifact overlaid). Over 8 months, all depressive symptoms had vanished, except a slight sensory overload in vibrant situations.

## Discussion

The present case study describes a patient most likely presenting with “autoimmune depression” in the context of multiple sclerosis with typical MRI and CSF pathologies, with mild concomitant latent systemic autoimmune response, unknown antineuronal antibodies in the CSF, and a good response to immunosuppressive treatment.

### Diagnostic Considerations

MRI images with several supratentorial WM lesions and inflammatory CSF syndrome with OCBs and transient local IgM synthesis are compatible with multiple sclerosis. When evaluating the clinical episode of ~20 years ago, the patient had experienced one clinical attack compatible with an episode of multiple sclerosis. The detection of OCBs replaces dissemination in time, according to the 2017 revised McDonald criteria (16); therefore, multiple sclerosis was diagnosed. Thus, immunosuppressive treatment of the underlying multiple sclerosis could also indirectly improve depression. In order to diagnose multiple sclerosis, more likely differential diagnoses may need to be excluded. The high ANA titers in combination with lymphopenia and brain involvement could indicate connective tissue disease. However, ENA differentiation and anti-ds-DNA antibodies remained unremarkable. Therefore, the criteria of the American College of Rheumatology (ACR) and Systemic Lupus Collaborating Clinics (SLICC) for SLE were not fulfilled [only three (brain involvement, lymphopenia, ANAs) out of four required ACR criteria, and three (brain involvement, lymphopenia, ANAs) of four required SLICC criteria were fulfilled) (17, 18). In addition, an axo-dendritic anti-neuronal autoantibody signal (which is not to be expected in healthy controls and cannot be typically observed in patients with multiple sclerosis, according to experiences in the Department of Neurology and Experimental Neurology, Charité Berlin from HP) was detected in indirect immunofluorescence on unfixed murine brain tissue on many neurons, especially not only on granule cells in the cerebellum, hippocampus, and olfactory bulb, but also in the corpus callosum. It raises the possibility of additive effects of antineuronal antibodies (which can be found in patients with autoimmune encephalitis) causing depressive symptoms. However, the criteria of possible autoimmune encephalitis were not fulfilled due to the lack of subacute onset (19).

### Clinical Significance

Irrespective of the discussed differential diagnostic considerations, the presented case shows that an autoimmune pathophysiology could hide behind a classical manifestation of a depressive syndrome. It is well known that fatigue symptoms can occur in patients with MS. However, the symptoms in the presented patient clearly go beyond a fatigue symptomatology. Thus, the case demonstrates the importance of extended diagnostics also for patients with classic depressive syndromes. Clinical signs pointing to autoimmune depression in our case were the poor response to a classical treatment and the MRI and CSF abnormalities. The testing of CSF on unfixed mouse brain slices could finally shed light on associated autoantibodies. The subsequent immunosuppressive therapy has to be regarded as a more causal treatment of the psychiatric disorder and rapid clinical improvement validated the whole concept. The individual maintenance treatment with methotrexate might also have helped to prevent the progression of the disease. The frequency of such cases in clinical samples with depressive disorder is largely unclear. However, CSF studies reveal that 6.5% of depressive patients receiving CSF analysis display OCBs (20). This observation suggests that similar cases might occur more frequently than previously thought.

### Limitations

The functional relevance of the antineuronal autoantibodies and the exact epitope target remain unclear, reflecting the still early development of this approach in psychiatry. In future similar cases, a scientific analysis of the detected autoantibodies would be useful if the patients agree to such studies. This could increase our understanding of the additional effect of the autoantibodies. Even a purely comorbid presence of a depressive disease in an independently existing multiple sclerosis seems possible; following these considerations, an antidepressant effect of corticosteroids without any anti-inflammatory effects would be conceivable. However, in the authors’ view—in consideration of the findings of inflammatory CSF and MRI changes, the antineuronal antibodies, poor response to classic antidepressant medication, and rapid and sustained improvement under anti-inflammatory treatment—it seems more likely that the response to corticosteroids is caused by an anti-inflammatory effect from the corticosteroids. Due to stable multiple sclerosis signs (for over 20 years) and the possible overlap with a systemic autoimmune disease, individual maintenance therapy with methotrexate was started first. At the time of publication, the beginning of a classical relapse prophylaxis for multiple sclerosis (e.g., with teriflunomide) to avoid the development of progressive multiple sclerosis was recommended to the patient. He also had vitamin B12 and folic acid deficiencies. A depressive disorder that was either caused or aggravated by this cannot be excluded. Notably, substitution initially did not lead to any improvement. Only anti-inflammatory treatment led to a strong and sustained improvement of the depressive symptoms; therefore, we do not assume that the symptoms were caused by the vitamin deficiency alone.

## Conclusions

This case report describes a patient presenting with probable autoimmune depression in the context of multiple sclerosis with additional antineuronal autoantibodies with a yet unspecified target epitope, which was detected by a tissue test producing an axo-dendritic signal in several brain regions. The importance of autoantibodies in such constellations requires further investigation. The detection of organic causes of psychiatric disorders is of high clinical relevance because it enables alternative and more causal treatment approaches, as demonstrated in the current case report.

## Data Availability Statement

All necessary information is mentioned in the article.

## Ethics Statement

The patient has given his written informed consent for this case report, including the presented images, to be published.

## Author Contributions

DE, PS, SR, and LT treated the patient. DE performed the data research and wrote the paper. MM supported the data collection. SR, RD and HP performed the neurological interpretation. SR and RD performed the CSF basic analyses, HP performed the tissue testing. NV performed the rheumatological tests and immunological interpretation. KE performed and interpreted the MRIs. KR, BLF, KN, SM, and KD supported the clinical and laboratory interpretation. All authors were critically involved in the theoretical discussion and composition of the manuscript. All authors contributed to the article and approved the submitted version.

## Funding

The article processing charge was funded by the German Research Foundation (DFG) and the University of Freiburg in the funding program Open Access Publishing.

## Conflict of Interest

SR: Receiving consulting and lecture fees, grant and research support from Bayer Vital, Biogen, Merck Serono, Novartis, Sanofi-Aventis, Genzyme, Roche and Teva. Furthermore, SR indicates that he is a founding executive board member of ravo Diagnostika GmbH Freiburg. RD: Receiving lecture fees from Roche and travel grants from Biogen. KD: Steering Committee Neurosciences, Janssen. LT: Advisory boards, lectures, or travel grants within the last three years: Roche, Eli Lilly, Janssen-Cilag, Novartis, Shire, UCB, GSK, Servier, Janssen and Cyberonics.

The remaining authors declare that the research was conducted in the absence of any commercial or financial relationships that could be construed as a potential conflict of interest.
